# Oral Surgery Procedures in a Patient Affected by Hereditary Angioedema Type I

**DOI:** 10.1155/2022/6602411

**Published:** 2022-01-29

**Authors:** Chiara Cinquini, Simonetta Santarelli, Alberto Marianelli, Marco Nisi, Mario Gabriele, Antonio Barone

**Affiliations:** Department of Surgical, Medical, Molecular and of Critical Area Pathologies, Complex Operative Unit of Stomatology and Oral Surgery, University-Hospital of Pisa, University of Pisa, 56126 Pisa, Italy

## Abstract

Hereditary Angioedema (HAE) is a rare disease characterized by a deficiency or a reduced function of the plasma protein C1 esterase inhibitor (C1-INH), which is involved in the downregulation of several inflammatory pathways. Patients affected by HAE suffer from episodic swellings of subcutaneous or submucosal tissues. Swellings can be caused by stress or dental and surgical procedures and can be life-threatening if the airways are involved. We have reported a clinical case of a patient affected by HAE type I who underwent oral surgery procedures under a short-term prophylaxis with C1-INH plasma-derived concentrate. The patient underwent a cyst removal, multiple tooth extractions, and an excisional biopsy with a prophylaxis with C1-INH plasma-derived concentrate and was hospitalized for 36 hours after the surgery to be monitored for possible HAE attacks. During the hospitalization, the patient did not show signs of swelling nor of HAE attacks. At 14 and 28 days after the surgery, the patient presented a good surgical healing. The prophylactic intravenous infusion of C1-INH concentrate was successful in preventing acute HAE attacks after oral surgery procedures.

## 1. Introduction

Hereditary angioedema (HAE) is a rare autosomal dominant disease characterized by a deficiency or a reduced function of the C1 esterase inhibitor (C1-INH) [1], a plasma protein belonging to the serine protease inhibitors encoded by the SERPING 1 gene [2]. Its incidence ranges from 1/50.000 to 1/100.000, with no distinction of race and sex [3]. Three variants of HAE are portrayed in scientific literature: type I affects 85% of patients and presents low plasma levels of C1-INH, and type II affects 15% of patients and is characterized by inactive C1-INH with normal plasma levels [4]. HAE with normal C1-INH (HAE-nC1INH) is rare and is characterized by normal C1-INH level and function and may be associated with mutations in the coagulation factor XII gene or in other known and unknown genes [5, 6].

C1 esterase inhibitor (C1-INH) is involved in the regulation of several inflammatory pathways [1]. Mutations in the gene encoding for the protein C1-INH (SERPING 1 gene) might prevent the synthesis of the protein (HAE type I) or produce a nonfunctioning protein (HAE type II) and both mutations lead to the failure to control the local production of bradykinin. The massive release of this inflammatory mediator increases vascular permeability, thus causing consequent edema of the anatomical areas involved [7]. HAE diagnosis may be suggested by recurrent episodes of swelling and by the presence of abdominal pain [8], even though the final diagnosis can be confirmed only through serological tests [9].

HAE type I is characterized by low plasma levels of C1-INH associated with a reduced function and reduced levels of a protein, called complement component 4 (C4), which is a protein belonging to the complement system [10].

Some authors showed that the key factors indicating high clinical suspicion for hereditary angioedema are family history and early age in onset of symptoms [8].

Patients affected by HAE suffer from episodic swellings affecting the subcutaneous and submucosal tissues of hands, feet, face, genitals, or gastrointestinal tract [11]; these acute onsets can be life-threatening when the upper airways are involved, especially the larynx, which can cause asphyxia. HAE attacks can be triggered by physical or psychological stress or can arise spontaneously; local trauma such as dental treatments and routine oral surgery procedures may increase the risk of HAE attacks within 4-48 hours from the intervention [12], typically starting near the area of trauma [9]. Several cases of patients' death after dental procedures are described in literature, involving not exclusively patients with recurrent HAE attacks. The mortality rate after oral surgery procedures without adequate prophylaxis was set at 30-40% in the past decades [13]; at the present time, short-term prophylaxis is used to prevent possible HAE attacks following surgical procedures or other possible triggers [14]. Throughout the years, a considerable number of drugs have been used for prophylaxis before surgical procedures such as C1-INH concentrate, attenuated androgenic hormone therapy, episolon aminocaproic acid, and fresh frozen plasma [12]; C1-INH concentrate has been the most efficient medication because it was developed focusing on the origins of HAE [7].

C1-INH concentrate (plasma-derived or recombinant) has been successfully used to treat acute HAE attacks for many years and, from 2013, has been approved in Europe for short-term prophylaxis before dental or surgical procedures in patients affected by HAE type I [14]. C1-INH concentrate has a long half-life and can persist in the blood stream for 1-2 days after the intravenous infusion administration [13].

Other medications approved for the treatment of acute HAE attacks are the kallikrein inhibitors (Ecallantide) and the bradykinin B2 receptor antagonists (Icatibant) [15, 16].

Hereditary angioedema is a rare disease that requires special precautions even for common dental or surgical procedures because the consequences might be fatal.

An adjunctive issue is the frequent delayed diagnosis of HAE [17], leading to the effective possibility of having to deal with patients affected by HAE and without being aware of the disease.

This report describes the management of oral surgical intervention in a patient affected by HAE type I.

## 2. Case Report

A 37-year-old man referred to the Unit of Oral Surgery because of episodic swellings in the inferior incisive area. The patient received a complete intraoral examination, showing a complete dentition and a pedunculate lesion of the right buccal mucosa.

**Figure 1:**
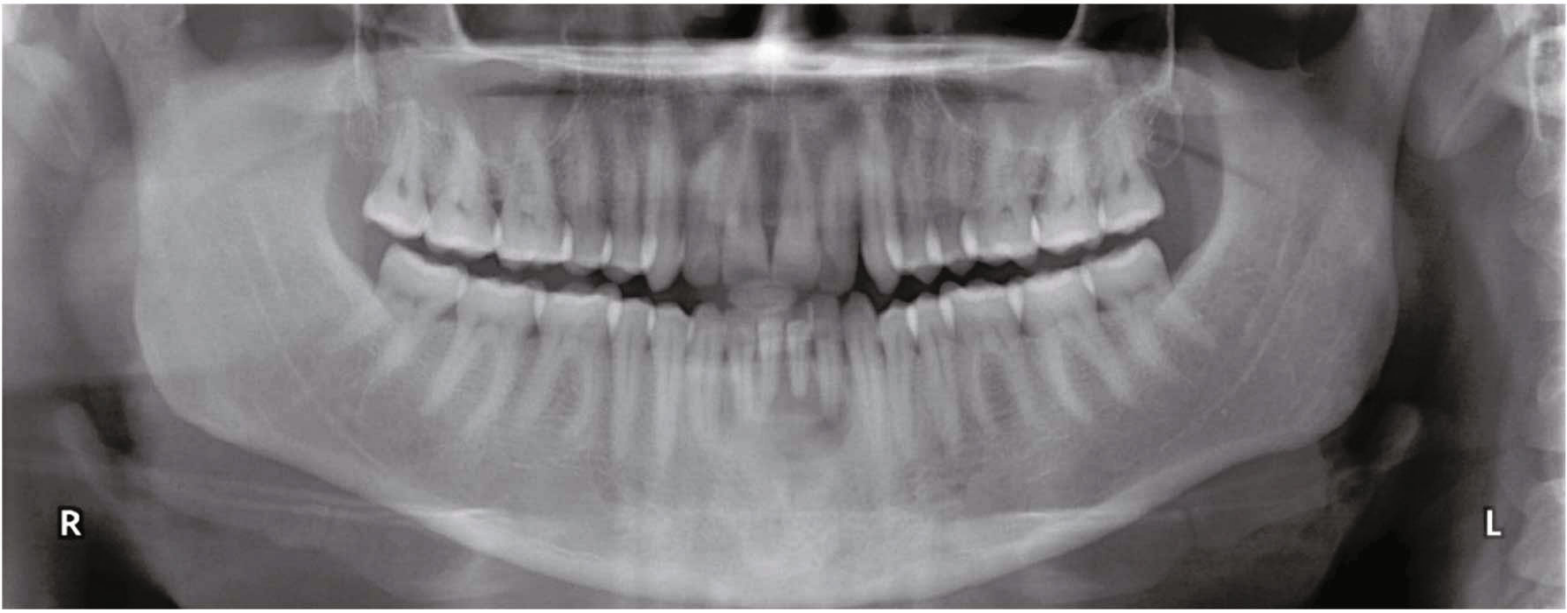
Orthopantomogram showing the mandibular radiolucent area.

During the clinical examination, a diffuse mild swelling was detected at the mandibular buccal incisive area; an orthopantomogram was then prescribed to further investigate the possible origin of the swelling. The radiographic examination revealed a unilocular radiolucency with thin sclerotic margins adjacent to the apical area of mandibular incisors (Figure 1).

Pulp vitality test was negative for teeth 3.1 and 3.2 (lower left incisors), and the teeth were sensitive to percussion. Moreover, teeth 4.1, 3.1, and 3.2 showed a grade III mobility. The suspected diagnosis was inflammatory odontogenic cyst, and the planned surgical intervention was the enucleation of the osteolytic lesion and the evaluation of the involved teeth. Moreover, the biopsy of the pedunculate lesion of the right buccal mucosa was scheduled.

The patient's medical history was collected: since the age of 14, he suffered from four episodes of swelling per year, localized in the hands, feet, face, and genitals. During these episodes, the swelling occurred slowly, involving only one anatomical site. It was associated with a nonpruritic rash and absence of fever, which gradually reduced during the following 3-4 days. In the past years, the patient had been hospitalized many times for episodes of abdominal pain and diarrhea. The hematologic analysis for suspected HAE was performed in December 2018: the C1-INH plasmatic concentration was 7.8 mg/dl (with a normal range between 12 and 30), the functional C1-INH was 34% (with a normal range between 30 and 130), and C4 was 7.7 mg/dl (with a normal range between 10 and 40), confirming the diagnosis of HAE type I.

The medical family history was positive; indeed, his brother, diagnosed with HAE type I, had died from asphyxia due to an HAE attack which occurred after a dental extraction. Complete hematologic analysis was requested a few days before the surgical procedure; erythrocyte, leucocyte, platelet count, prothrombin activity, aPTT and fibrinogen, and coagulation factors were within a normal range.

In agreement with the Immuno-allergology Department, the patient underwent the surgical procedure with a prophylaxis with C1-INH plasma-derived concentrate (Berinert). Berinert is a C1-INH plasma-derived concentrate, usually administered for the short-term prophylaxis of HAE attacks before surgical procedures. The recommended dose of Berinert is 20 IU/kg (international unit/kilogram), depending on the body weight. On the basis of the patient's weight, 1500 IU were slowly infused intravenously 1 hour before the surgery.

**Figure 2:**
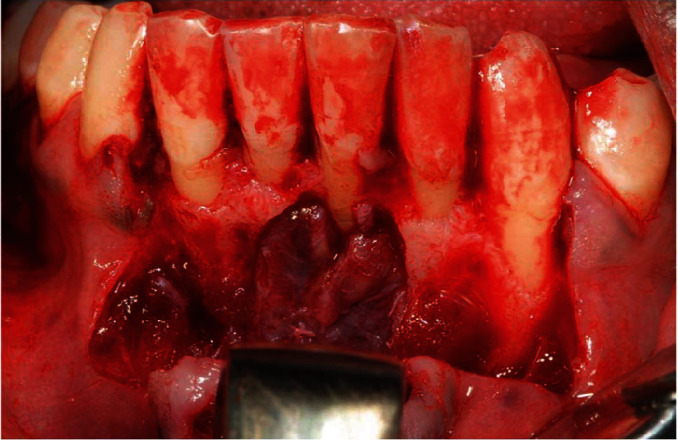
Flap elevation and cyst exposure.

**Figure 3:**
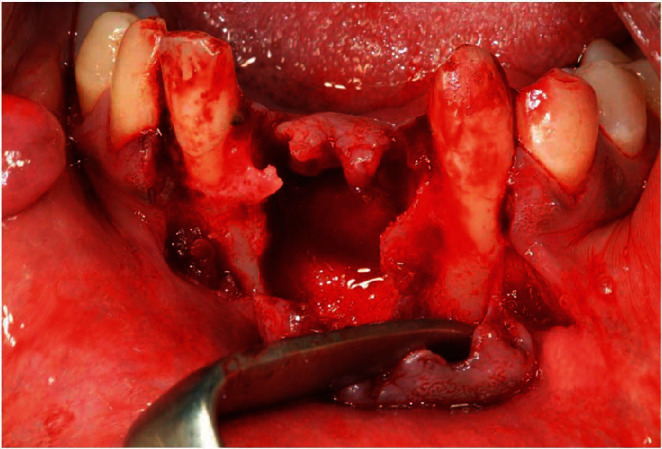
Bone defect after tooth extractions and cyst removal.

All the surgical procedures were performed under local anesthesia with lidocaine, while monitoring the vital signs (electrocardiography, hearth-rate, blood pressure, and SpO_2_). A mucoperiosteal flap was elevated in the central area of the mandible, revealing a mandibular cyst of approximately 1.5 × 1.2 cm. The cyst was surgically excised, and three mandibular incisors (4.1, 3.1, and 3.2) were extracted due to the extensive bone loss and high mobility (Figures 2 and 3); in fact, both lingual and buccal cortical plates were completely absent once the cyst was removed and the teeth showed a grade III mobility, resulting in an endodontic-periodontal lesion with a hopeless prognosis. The preservation of these teeth could have been resulted in the persistent presence of infection and inflammation, which should be avoided in a HAE patient [4]. An excisional biopsy was also performed to remove and analyze the pedunculate lesion of the right buccal mucosa.

**Figure 4:**
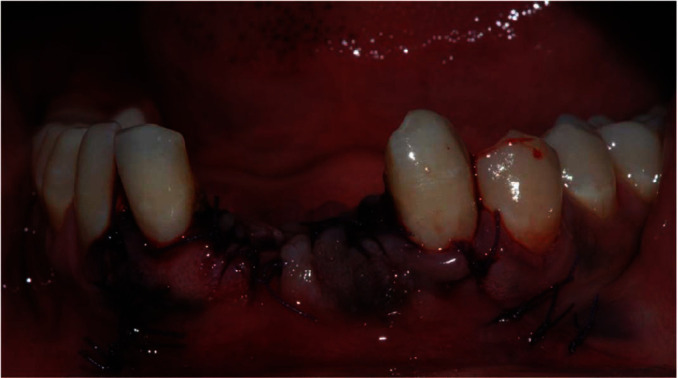
Clinical image immediately after the surgery.

Absorbable sutures were placed to avoid postsurgical removal (Figure 4). Moreover, these sutures made of copolymers of glycolide and lactide are broken down by hydrolysis processes and are not associated with a strong inflammatory reaction [18]. Both surgical specimens were delivered to the Pathological Anatomy Laboratory for the histopathological evaluation. Ropivacaine was instilled in the surgical wound immediately after the surgery to reduce postoperative pain and the consequent stress for the patient.

After the surgery, the patient was asked to report pain using the Numeric Rating Score (NRS) from 0 (no pain at all) to 10 (worst imaginable pain); the NRS value assessed was 6, and the patient was provided with Tramadol 100 mg administered intravenously. A postsurgical antibiotic prophylaxis with Clarithromycin was prescribed at the dosage of 500 mg (one tablet) every 12 hours for seven days. Beta-lactam antibiotics such as amoxicillin were avoided since they may cause drug-induced angioedema [19].

The patient was hospitalized for 36 hours after the surgery to be monitored for possible HAE attacks.

After 36 hours of hospitalization, the patient showed absence of acute HAE episode and he was discharged. He was given exhaustive postsurgical instructions and provided with Icatibant (Firazyr), an antagonist of bradykinin B2 receptors, to be administered subcutaneously by the patient himself in case of an acute attack after the hospitalization period. He was also given the recommendation to go to the emergency department in case of any suspected HAE attacks.

The patient was reevaluated 7 days after the surgery. He had had no occurrence of swelling in the past days and had not required to access the emergency department.

No relevant surgical-related edema was observed. The patient reported no pain (NRS = 0) and no reduction in mouth opening. At the clinical examination, no suppuration or local signs of inflammation were evident.

**Figure 5:**
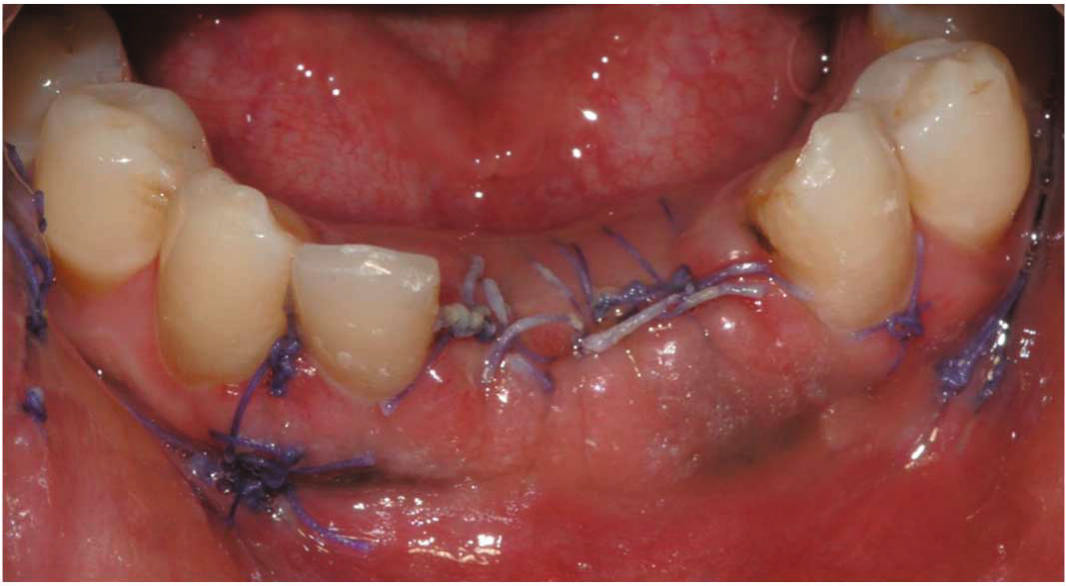
Clinical image after 14 days from the surgery.

At 14 (Figure 5) and 28 days after the surgery, the patient presented a good surgical healing and an excellent oral hygiene.

The histopathological findings of the intrabony lesion showed the presence of a fibrous wall with a lymphoplasmacytic and monocytic infiltrate, and a lining epithelium, confirming the clinical diagnosis of inflammatory odontogenic cyst (radicular cyst). With regard to the pedunculate lesion of the buccal mucosa, the presence of sclerotic connective tissue with signs of acanthosis and parakeratosis was compatible with a local fibrous hyperplasia on an irritative basis.

Special attention will be paid for future prosthetic rehabilitation since in literature; there are described acute HAE attacks following impressions for prosthetic rehabilitations [20].

## 3. Discussion

In the past years, the administration of preoperative fresh frozen plasma (FFP) has been an effective method to prevent HAE attacks following dental or surgical procedures [21].

The use of C1-INH concentrate was approved in 2009 in Europe and in 2010 in the USA for the treatment of acute HAE attacks, while its use as prophylactic treatment was finally approved in Europe in 2013.

Many studies in literature reported the use of C1-INH concentrate as prophylaxis before surgical procedures, showing good outcomes.

Maeda et al. [7] described a case report of a patient affected by HAE type I who suffered from swellings after the injection of local anesthetics for routinary dental treatments. The patient required the extraction of a lower third molar, and he was treated under local anesthesia; the infusion of C1-INH was performed just after the surgical procedure. In this case, no postprocedural swelling was observed.

Sanuki et al. [22] reported a successful management of multiple tooth extractions in a patient affected by HAE with the preprocedural administration of the C1-INH concentrate. The postoperative period was uneventful, and the patient was discharged home 3 days after the surgery.

A retrospective study on the efficacy of C1-INH concentrate prophylaxis in preventing HAE attacks showed that out of 24 patients who underwent invasive surgical procedures (varying from dental extractions to aortic aneurysm repair), none of them had a HAE attack [14].

Another retrospective study performed on 24 HAE patients who underwent 66 dental procedures (ranging from dental extraction to orthodontic treatment) showed the efficacy of a preprocedural prophylaxis with C1-INH concentrate or treatment with attenuated androgens (AAs) [11]. According to the authors, only 3 patients experienced a mild upper airway edema; none of these patients received a preprocedural prophylaxis with C1-INH or a maintenance treatment with AAs.

A larger study including 91 patients suffering from HAE showed that 98% of the patients, who were treated with C1-INH concentrate as prophylaxis, had an uneventful healing period after surgical procedures [23].

The International WAO/EAACI (World Allergy Organization and the European Academy of Allergy and Clinical Immunology) guidelines suggest the use of the C1-INH concentrate as presurgical prophylaxis in patients affected by HAE [10].

The importance of presurgical prophylaxis is confirmed by the fact that HAE attacks do not respond to many antiedema treatments such as antihistamines, corticosteroids, and adrenaline [24].

The prevention of acute attacks is the best course of action in cases of surgical procedures for patients affected by HAE, especially considering the fact that the treatment of acute attacks may be difficult and a potentially life-threatening complication. However, it should be remembered that acute HAE attacks may occur after relatively minor surgical procedures despite the presurgical prophylaxis [25]; for this reason, patients should be carefully monitored and instructed on what to do at home in case of an acute attack, according to what is reported in the case report section.

In the present case, a presurgical prophylaxis with C1-INH concentrate was effective in preventing acute HAE attacks and the postsurgical period was uneventful.

## Supplementary Materials

Supplementary Materials Supplementary file 1: care checklist.
